# Molecular identification of *Oesophagostomum* spp. from ‘village’ chimpanzees in Uganda and their phylogenetic relationship with those of other primates

**DOI:** 10.1098/rsos.150471

**Published:** 2015-11-11

**Authors:** Narumi Ota, Hideo Hasegawa, Matthew R. McLennan, Takanori Kooriyama, Hiroshi Sato, Paula A. Pebsworth, Michael A. Huffman

**Affiliations:** 1 Department of Biology, Faculty of Medicine, Oita University, 1-1 Idaigaoka, Hasama, Yufu, Oita 879-5593, Japan; 2 Faculty of Humanities and Social Sciences, Anthropology Centre for Conservation, Environment and Development, Oxford Brookes University, Gipsy Lane Campus, Oxford OX3 0BP, UK; 3 Department of Veterinary Science, School of Veterinary Medicine, Rakuno Gakuen University, 582, Bunkyodai-Midori, Ebetsu, Hokkaido 069-8501, Japan; 4 Laboratory of Parasitology, Joint Faculty of Veterinary Medicine, Yamaguchi University, 1677-1 Yoshida, Yamaguchi 7s53-8515, Japan; 5 Department of Anthropology, The University of Texas, San Antonio, TX 78249, USA; 6 Department of Soil Science, Stellenbosch University, Stellenbosch, South Africa; 7 Section of Social Systems Evolution, Primate Research Institute, Kyoto University, 41-2, Kanrin, Inuyama, Aichi 484-8506, Japan

**Keywords:** DNA sequencing, nodular worms, *Pan troglodytes*, *Papio* spp., phylogeny, *Macaca fuscata*

## Abstract

*Oesophagostomum* spp. are parasitic nematodes of mammals, including humans and other primates. To identify species and determine phylogeny, we analysed DNA sequences of adult and larval *Oesophagostomum* from wild chimpanzees in Bulindi, Uganda, which inhabit degraded forest fragments amid villages. Oesophagostome larvae and/or eggs from baboons in Tanzania and South Africa and from a Japanese macaque were also sequenced. Based on the internal transcribed spacer 2 (ITS2) of nuclear ribosomal DNA and partial cytochrome *c* oxidase subunit 1 gene (*Cox1*) of mtDNA, *O. stephanostomum* and *O. bifurcum* were identified from chimpanzees. Bulindi is the second locality where molecular detection of *O. bifurcum* in wild chimpanzees has been made. While most *O. stephanostomum* had ITS2 genotypes recorded previously, three new genotypes were detected. Among four ITS2 genotypes of *O. bifurcum* from chimpanzees, one was identical to that from various monkey species in Kibale, Uganda, and baboons from Tanzania and South Africa; another was shared by a baboon from Tanzania. No genotype was identical with that of the cryptic species reported from humans and monkeys in Kibale. Phylogeny based on *Cox1* sequences of *O. stephanostomum* showed locality-dependent clades, whereas those of *O. bifurcum* formed clades composed of worms from different hosts and localities.

## 1. Background

Nodular worms, *Oesophagostomum* spp., are intestinal nematodes of mammals, especially pigs, ruminants and primates. Some are potentially pathogenic to livestock, whereas several species are known to cause human infections [1]. *Oesophagostomum bifurcum* is of major human health concern in focally endemic areas of Africa, specifically Ghana and Togo in West Africa, where prevalence can be high [2–4]. The infections of these species can cause serious clinical disease (oesophagostomiasis) associated with formation of nodular lesions or abscesses in the intestinal wall of humans and non-human primates [1,2,5]. The potential for transmission of oesophagostomes between humans and non-human primates under natural conditions has been the source of considerable debate. Initially, human *O. bifurcum* infections were considered a rare zoonosis [6]. However, molecular evidence later showed that *O. bifurcum* in humans in northern Ghana was distinct from that in several species of monkey [7], suggesting human oesophagostomiasis there was not a zoonotic infection. Meanwhile, *O. stephanostomum* and a cryptic oesophagostome species were recently demonstrated molecularly from sympatric humans and non-human primates in Uganda, suggesting occurrence of zoonotic transmission [8,9]. Transmission of oesophagostomes between humans and non-human primates could potentially occur in areas where hosts’ habitats are overlapped. Moreover, transmission could be enhanced where humans and great apes share the same habitat, because they are genetically more similar to each other than to other sympatric primate hosts [10].

Across tropical Africa, great ape habitats are being transformed by human activities, including agriculture, mining and logging. Consequently, apes increasingly inhabit disturbed environments near people [11], with potential implications for both great ape conservation and public health. In Bulindi, Uganda, East African chimpanzees (*Pan troglodytes schweinfurthii*) live in exceptionally close contact with humans [12]; thus, risk of disease transmission is potentially high at this site [13]. *Oesophagostomum* is a common parasite of wild chimpanzees. While infections appear to have little effect on the health of chimpanzee hosts in most cases, severe clinical signs of oesophagostomiasis in wild chimpanzees have been reported [14–16]. Moreover, *Oesophagostomum* is the primary target of anti-parasite behaviours recorded in detail for chimpanzees [17], including at Bulindi [13]. Therefore, this parasite is both a chimpanzee and human health concern [10]. A previous morphological examination of adult worms found in chimpanzee faeces from Bulindi in 2007 indicated that these chimpanzees were infected with more than one form or species of *Oesophagostomum* [13]. Thus, the objective of this study was to identify the species of oesophagostomes in the chimpanzees of Bulindi using DNA sequence analysis. We then sought to determine their phylogenetic relationship to oesophagostome isolates described in other primates, including humans. Oesophagostome isolates from African baboons and Asian macaques were additionally sequenced for this study. We evaluate our findings in the context of on-going debates concerning the zoonotic potential of *Oesophagostomum* in areas of close human–non-human primate sympatry.

### 1.1 Area surveyed

Bulindi Parish is situated in the Hoima District of western Uganda between 1°28′ N and 31°28′ E. Hoima District is notable as a region of exceptionally close sympatry between multiple groups (‘communities’) of wild chimpanzees and a fast-growing human farming population [18]. Human population density in the district was estimated at 159 persons per km^2^ in 2014 [19]. A recent genetic census revealed that a population of 256–319 chimpanzees inhabit this human-dominated landscape at a density of *ca* 0.4 individuals per km^2^, across an area of more than 600 km^2^ [20]. Like chimpanzee communities elsewhere in Hoima, chimpanzees in the Bulindi area inhabit small, degraded riparian forest fragments amid farmland and villages [21,22], and enter fields and village areas regularly to feed on agricultural crops [23]. The Bulindi community comprised 19 individuals during the period considered here, including four mature males (three adult, one subadult), seven mature females (six adult, one subadult) and eight juveniles and infants, and ranged over an area of *ca* 20 km^2^. Chimpanzees in Bulindi are sympatric with four other species of diurnal non-human primate: olive baboons (*Papio anubis*), tantalus monkeys (*Chlorocebus tantalus*), black and white colobus monkeys (*Colobus guereza*) and blue monkeys (*Cercopithecus mitis*).

## 2. Material and methods

### 2.1 Sample collection

Chimpanzee faecal samples were collected by M.R.M. during September–November 2012 and February–April 2013 (*n*=406). Chimpanzees at Bulindi were not habituated to close observation during this study; thus, faeces were collected anonymously at chimpanzee nest sites and during tracking. Faecal samples were collected during morning hours within 6 h of defecation. Faeces were examined macroscopically and adult worms observed were fixed and stored in more than 99% ethanol. Samples collected in April 2013 (*n*=38) were subjected to the modified Harada–Mori filter paper culture [24], irrespective of presence or absence of adult worms. After 7–14 days, the bottom water was checked with a magnifying glass, and larva-positive water was transferred to a 5 ml serum tube using a disposable plastic pipette, fixed and stored in more than 99% ethanol. All samples were transported to the Department of Biology, Faculty of Medicine, Oita University, Japan, for further analysis. Besides the samples from Uganda chimpanzees, filariform larvae reared from the collected faeces of two yellow baboons (*Papio cynocephalus*) of Mahale, Tanzania (see [25,26]), and one Japanese macaque (*Macaca fuscata*) from Oita, Japan, and eggs isolated from faeces of two chacma baboons (*Papio ursinus*) of the Western Cape, South Africa (see [27]), were also analysed.

### 2.2 DNA extraction

From adult worms, a small piece of body was cut out using a sterilized scalpel blade and homogenized in 50 μl DW using a sterilized disposable plastic pestle, and 5 μl of the solution was mixed with 50 μl of liquid phase of DEXPAT^®^ (Takara Bio., Inc., Otsu, Shiga, Japan) in a sterilized 200 μl tube, and heated at 98°C for 30 min and then cooled on ice. For filariform larvae, the ethanol containing larvae was transferred to a sterilized disposable plastic dish and observed under a stereomicroscope to classify them based on the morphology [28]. Each filariform larva selected was picked up using a flame-sterilized fine insect needle attached to a glass rod, and washed in a drop of sterilized distilled water on a sterilized plastic dish. Then, the larva was cut using the flame-sterilized needle under a stereomicroscope, transferred using a 5 μl micropipette to 50 μl of liquid phase of DEXPAT^®^, and treated as in the case of adult worms. Eggs were collected from ethanol-fixed faecal solution under a stereomicroscope using a Pasteur pipette of which the distal end was burnt and pulled to form a fine capillary. Then, the eggs were washed in a drop of distilled water in a sterilized disposable plastic dish. Subsequently, eggs were transferred onto a small piece (3×3 mm) of cellophane on the dish using the pipette, covered with another piece of cellophane and crushed using the sterilized disposable plastic pestle. The cellophane pieces were put into 25 μl of liquid phase of DEXPAT^®^ in a 200 μl tube, and treated as in the case of adult or larva. The cooled solution was used as template.

### 2.3 DNA amplification

PCR was performed using polymerase KOD-Plus-Neo^®^ (Toyobo Co., Tokyo, Japan). Five microliters of template solution and primer sets were mixed with 50 μl prepared reaction solution. Primers used for amplification of the internal transcribed spacer (ITS) region were as follows: NC1 (forward: 5′-ACGTCTGGTTCAGGGTTGTT-3^′^), Civ18S1500F (forward: 5′-TTATTTCCCTTGAACGAGGAAT-3′), TW1 (forward: 5′-GTTTCCGTAGGTGAACCTGC-3′), AB28 (reverse: 5′-ATATGCTTAAGTTCAGCGGG T-3^′^) and NC2 (reverse: 5′-TTAGTTTCTTTTCCTCCGCT-3^′^) [29–31]. Primers used for amplification of mtDNA cytochrome *c* oxidase subunit 1 gene (*Cox1*) were as follows: StrCoxAfrF (forward: 5′-GTGGTTTTGGTAATTGAATGGTT-3^′^), OesoCoxF1 (forward: 5′-GTTTAAATAATTTAAGTTTT-3^′^), OesoCoxF4 (forward: 5′-AGATCTAATCATAAAGATAT-3^′^), JB3 (forward: 5′-TTTTTTGGGCATCCTGA GGTTTAT-3^′^); MH28R (reverse: 5′-CTAACTACATAATAAGTATCATG-3^′^) and JB4.5 (reverse: 5′-TA AAGAAAGAACATAATGAAAATG-3^′^) [25,32].

PCR condition for mtDNA *Cox1* was 94°C for 2 min; (98°C 15 s; 45°C 30 s; 68°C 30 s) × 30; (98°C 15 s; 55°C 30 s; 68°C 30 s) × 30; 68°C 5 min. For ITS, PCR condition was 94°C for 2 min; (98°C 10 s; 53°C 30 s; 68°C 30 s) × 30; (98°C 10 s; 58°C 30 s; 68°C 30 s) × 30; 68°C 5 min. When amplifications did not work adequately, the annealing temperature was lowered.

### 2.4 Sequencing

PCR products were mixed with EZ-Vision and electrophoresed in 1.5% agarose gel, and bands were excised from gel under a UV-illuminator. Then, DNA was extracted from the gel using a Nucleospin^®^ column (Macherey-Nagel Co., Düren, Germany), ethanol-precipitated and vacuum-dried. The purified products were processed using ABI BigDye Terminator^®^ v. 3.1 (Applied Biosystems, Foster City, CA) with one of the primers, and refined with Centri-Sep^®^ Spin Column (Princeton Separations Inc., Adelphia, NJ). Nucleotide sequences were determined using an ABI3130 sequencer (Applied Biosystems).

### 2.5 Phylogenetic analyses

Neighbour-joining (NJ) and maximum-likelihood (ML) algorithms were applied for the sequences using MEGA5 software [33,34]. Bootstrap confidence (1000 replicates) was also calculated using MEGA5. Sequences were aligned using ClustalW [35] if necessary. For analysis of sequences with single polymorphic locus, one of the bases was used. Sequences with plural polymorphic loci were not used in the analysis.

## 3. Results

Seventeen adult *Oesophagostomum* were found in 10 chimpanzee faecal samples (2.5% of 406 faeces inspected). Fourteen of these adults were measured (11 females, three males): median length was 20.5 mm (range: 18–25 mm). Of 38 faeces cultured, 22 (58%) were positive for *Oesophagostomum* larvae. All of the adults and 15 filariform larvae selected randomly from eight faecal cultures were subjected to DNA sequence analysis. ITS2 and *Cox1* sequences were successfully analysed for 19 and 27 worms, respectively. Both ITS2 and *Cox1* were sequenced for 15 worms (eight adults from eight faecal samples and six larvae from three faecal samples). Seven filariform larvae from yellow baboons of Tanzania, two filariform larvae from one Japanese macaque and two batches of eggs, with 10 and two eggs, from one chacma baboon of South Africa were tested and successfully sequenced for both ITS2 and *Cox1*. Only *Cox1* sequence was obtained from eggs isolated from the other chacma baboon individual.

**Figure 1.**
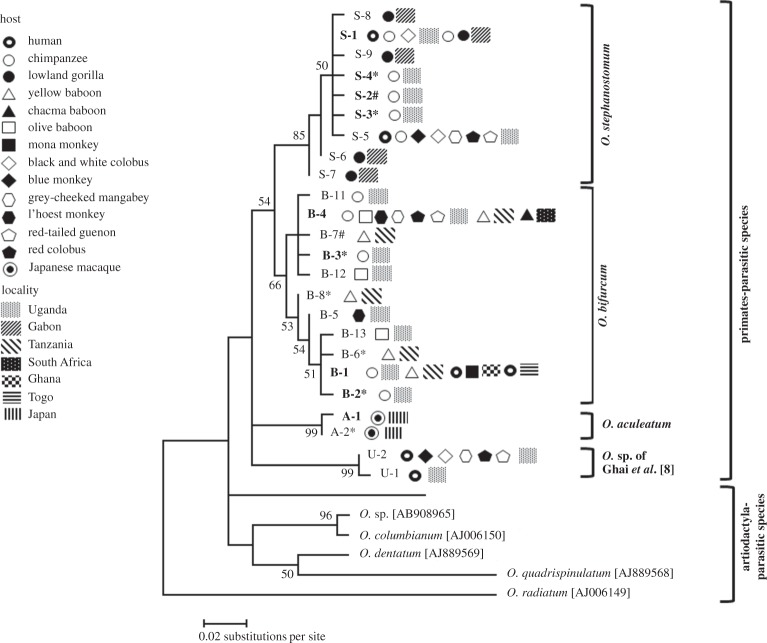
Phylogenetic analysis of *Oesophagostomum* spp. based on ITS2 sequences. The evolutionary history was inferred by using the maximum-likelihood method based on the Hasegawa-Kishino-Yano model [40]. The tree with the highest log likelihood (−872.8970) is shown. The tree is drawn to scale, with branch lengths measured in the number of substitutions per site. The analysis involved 30 nucleotide sequences. Sequences were aligned using ClustalW [35], and all positions containing gaps and missing data were eliminated. There were a total of 187 positions in the final dataset. The percentages of replicate trees in which the associated taxa clustered together in the bootstrap test (1000 replicates) are shown next to the branches [41], and values more than 50% are shown. * Newly found genotype. # Newly found genotype with polymorphic locus.

As shown in table 1 and figure 1, the ITS2 sequences obtained from the chimpanzees of Bulindi were classified into two groups, representing *O. stephanostomum* and *O. bifurcum*, respectively. All adult worms were proven to be *O. stephanostomum*, whereas a mixed infection with both species was found in two faecal samples: one sample contained adult *O. stephanostomum* but the larvae reared were *O. bifurcum*; the other gave larvae of both species by culture. Among the 15 worms of *O. stephanostomum* analysed successfully for ITS2, 12 had an identical ITS2 genotype (referred to as S-1 herein), which was the most common type of this species in western lowland gorillas (*Gorilla gorilla*) at Moukalaba-Doudou National Park in Gabon [36], but differed slightly from those found in chimpanzees and other primates found in Kibale, Uganda, by having one or two nucleotide substitutions [8] (S-5 in table 1 and figure 1). Meanwhile, the remaining three genotypes (from S-2 to S-4) are newly recorded, each having one nucleotide substitution, though the locus was polymorphic in S-2 (table 1).

**Table 1.** Comparison of nucleotide variation in the ITS2 region of rDNA of *Oesophagostomum* spp. collected in Bulindi, Uganda (boldface), and some other localities^1^. p.s., present study; n.a., not analysed.

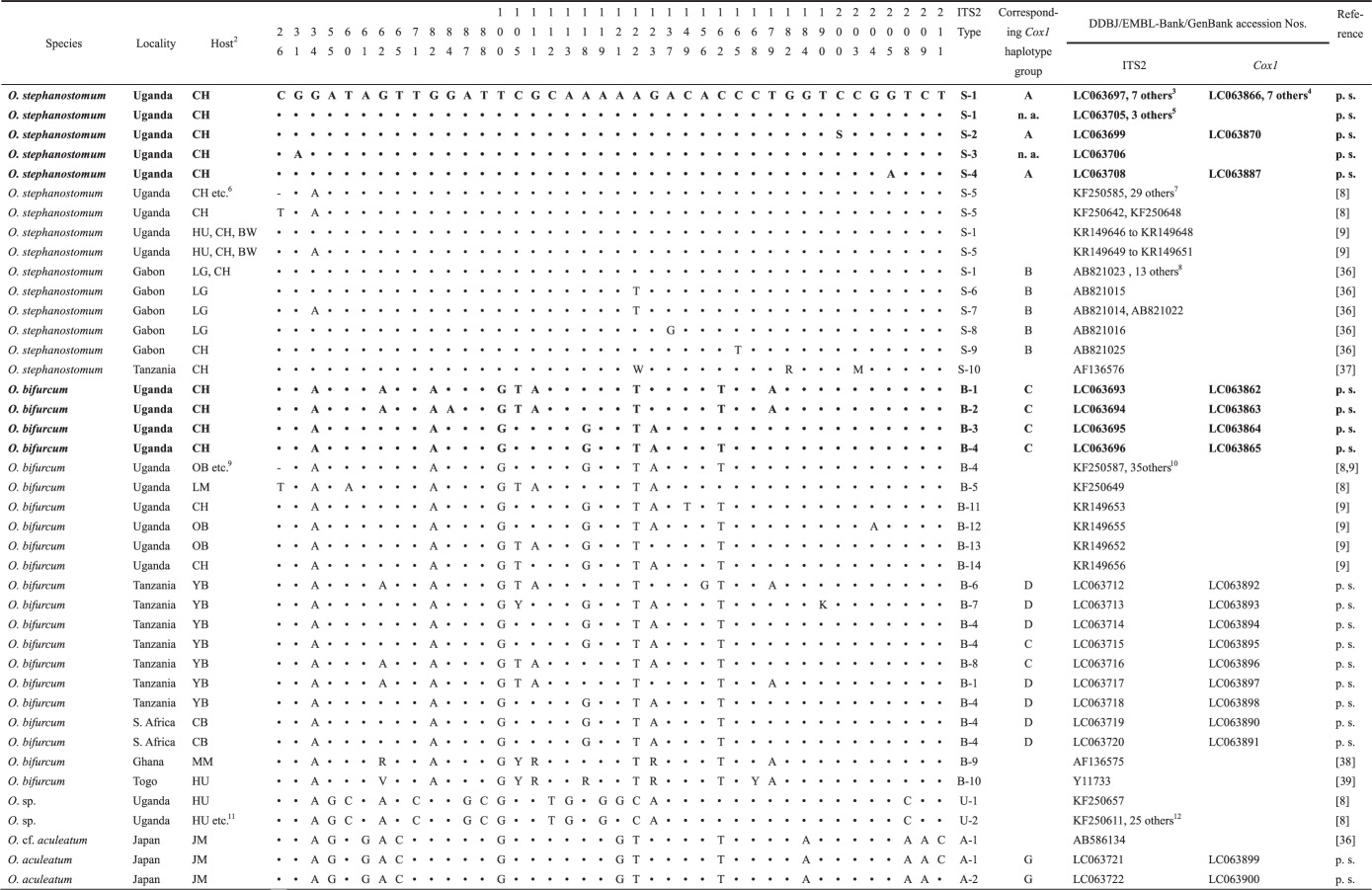

^1^Nucleotide position is expressed relative to the 5′-terminus of *O. stephanostomum* S-1 genotype. Dots denote an identical base to that of the uppermost sequence.

^2^Abbreviations of hosts: BM, blue monkey (*Cercopithecus mitis*); BW, black and white colobus (*Colobus guereza*); CB, chacma baboon (*Papio ursinus*); CH, chimpanzee (*Pan troglodytes*); GM, grey-cheeked mangabey (*Lophocebus albigena*); HU, human (*Homo sapiens*); JM, Japanese macaque (*Macaca fuscata*); LG, lowland gorilla (*Gorilla gorilla gorilla*); LM, l’hoest monkey (*Cercopithecus lhoesti*); MM, mona monkey (*Cercopithecus mona*); OB, olive baboon (*Papio anubis*); RC, red colobus (*Procolobus rufomitratus*); RT, red-tailed guenon (*Cercopithecus ascanius*); YB, yellow baboon (*Papio cynocephalus*).

^3^LC063698, LC063700-LC063704, LC063709.

^4^LC063867, LC063878-LC063882, LC063888.

^5^LC063707, LC063710, LC063711.

^6^BM, BW, GM, RC, RT.

^7^LM, GM, RC, RT.

^8^AB821013, AB821017–AB821021, AB821024–AB821030.

^9^GM, LM, RC, RT.

^10^KF250585–KF250588, KF250592, KF250594, KF250595, KF250598, KF250599, KF250605, KF250606, KF250612–KF250619, KF250631, KF250634, KF250635, KF250637–KF250644, KF250647, KF250648, KF250653, KR149654, KR149657.

^11^BM, BW, GM, RC, RT.

^12^KF250593, KF250597, KF250607, KF250608, KF250620–KF250625, KF250630, KF250632, KF250633, KF250636, KF250645, KF250646, KF250650–KF250652, KF250655-KF250660.

The four ITS2 sequences of *O. bifurcum* from the Bulindi chimpanzees differed slightly from each other. One genotype (B-4) was identical with that commonly found from those parasitic in various monkey species in Kibale [8], and also in yellow baboons of Tanzania and the chacma baboon of South Africa (table 1 and figure 1). Meanwhile, one (B-1) was shared by a yellow baboon of Tanzania and also by mona monkeys (*Cercopithecus mona*) and humans from Ghana and Togo (T-1 of Gasser *et al*. [38]; cf. Makouloutou *et al*. [36]; table 1 and figure 1). None of the present genotypes were identical or closely related to those of the cryptic species (*Oesophagostomum* sp. with accession numbers KF250611 and KF250655 [8]), which was demonstrated from humans and non-human primates in Kibale (table 1 and figure 1; genotypes U-1, U-2). Five of seven genotypes sequenced in this study were newly recorded.

**Figure 2.**
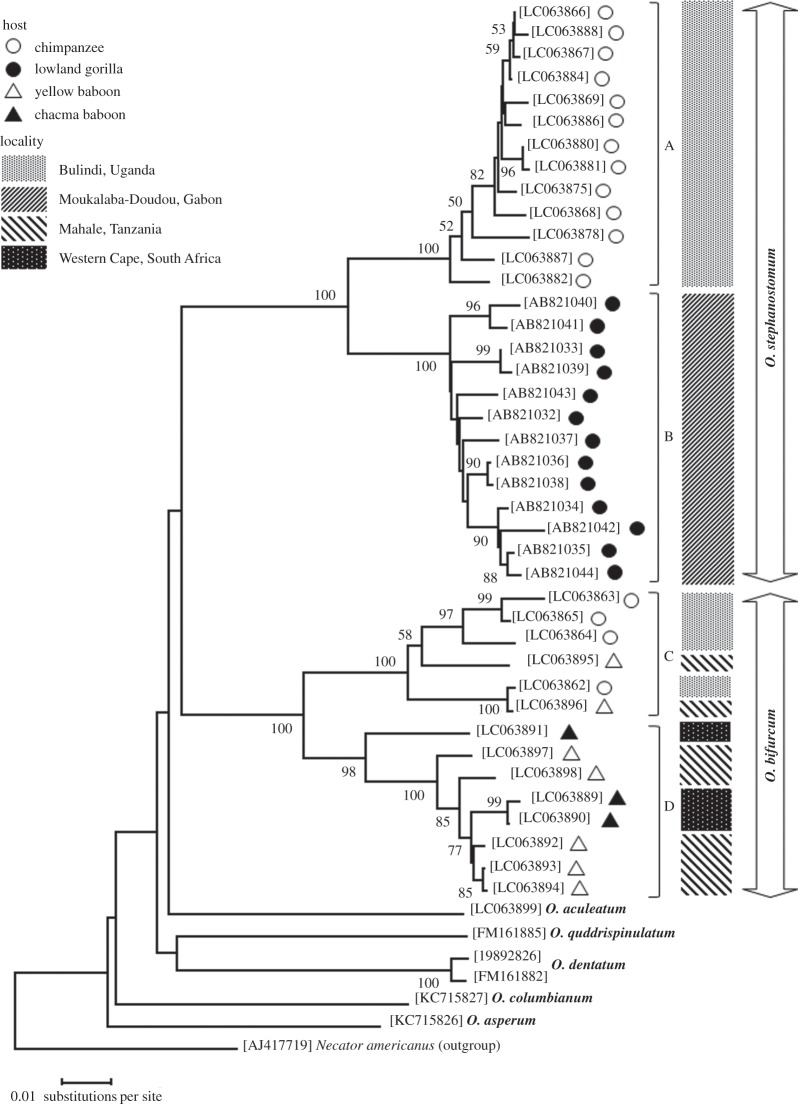
Evolutionary relationships of *Oesophagostomum* spp. inferred based on partial *Cox1* nucleotide sequences each with 766 bp using the neighbour-joining method [33]. The tree is drawn to scale, with branch lengths in the same units as those of the evolutionary distances used to infer the phylogenetic tree. The evolutionary distances were computed using the Kimura 2-parameter method [42] and are in the units of the number of base substitutions per site. The percentages of replicate trees in which the associated taxa clustered together in the bootstrap test (1000 replicates) are shown next to the branches [41].

Two sets of sequences of *Cox1* with 766 and 328 bp, respectively, of *Oesophagostomum* were tested for phylogenetic analysis. The longer sequence set included *O. stephanostomum* parasitic in great apes of Gabon besides those analysed in the present study; the shorter sequence set was formed by adding sequences of *O. bifurcum* from humans and mona monkeys from Ghana. NJ and ML analyses based on the nucleotides gave phylogenetic trees with similar topology, though bootstrap values were slightly lower in ML trees. By phylogenetic analyses on the longer sequences, *Cox1* of *O. stephanostomum*in the Bulindi chimpanzees formed a clear clade, which differed from the clade formed by those from gorillas in Gabon (figure 2; A and B). Meanwhile, *O. bifurcum* formed a somewhat intermingled topology (figure 2; C and D). Three haplotypes found in the parasitic worms in Bulindi chimpanzees were grouped, but the remaining one haplotype was closer to that in one yellow baboon of Tanzania. Five and three haplotypes of *O. bifurcum* parasitic in the yellow baboons of Tanzania and the chacma baboons of South Africa, respectively, formed another clade.

**Figure 3.**
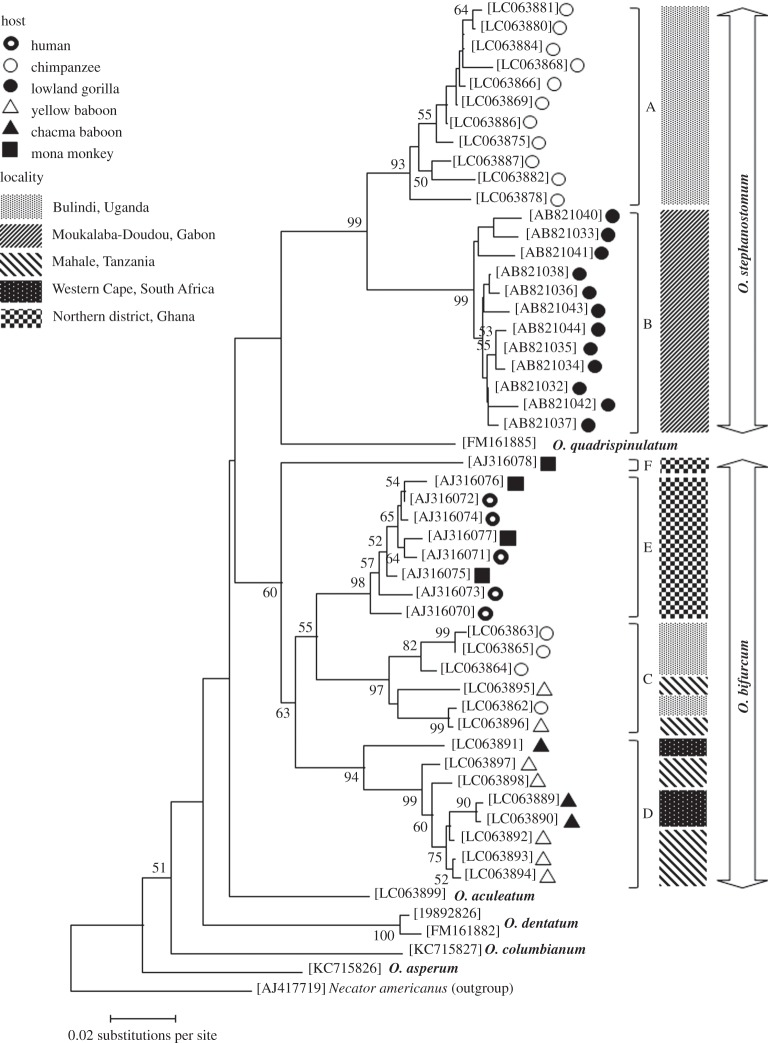
Evolutionary relationships of *Oesophagostomum* spp. inferred based on partial *Cox1* nucleotide sequences each with 328 bp using the neighbour-joining method [33]. The tree is drawn to scale, with branch lengths in the same units as those of the evolutionary distances used to infer the phylogenetic tree. The evolutionary distances were computed using the Kimura 2-parameter method [42] and are in the units of the number of base substitutions per site. The percentages of replicate trees in which the associated taxa clustered together in the bootstrap test (1000 replicates) are shown next to the branches [41].

From phylogenetic analyses of the shorter sequences, the phylogram obtained was largely identical with that on the longer sequences except that an additional clade corresponding to those of Ghana examples was formed in the *O. bifurcum* group (figure 3; E). One sequence from the mona monkey of Ghana was positioned outside of all other haplotype groups of *O. bifurcum* (figure 3; F).

## 4. Discussion

The present results demonstrate that chimpanzees in Bulindi, western Uganda were infected with both *O. stephanostomum* and *O. bifurcum*. Apparently, the former was the predominant species of this genus in chimpanzees as all 17 adults and 11 of 15 larvae were identified as *O. stephanostomum*. While we confirmed species by DNA analysis, adult worms measured in this study were longer (18–25 mm) than reported lengths of *O. bifurcum*, but within the normal range of *O. stephanostomum* [3]. Predominance of *O. stephanostomum* in chimpanzees is well documented [17,37]. Bulindi is the second locality where molecular detection of *O. bifurcum* has been made in wild chimpanzees. The presence of both species in Ugandan chimpanzees was reported previously by Krief *et al*. [10] and more recently by Cibot *et al*. [9], who also recorded mixed infections with the two species from chimpanzees at Kibale National Park, which is located about 180 km southwest of Bulindi. Because chimpanzees are closest to humans phylogenetically, the zoonotic potential of their oesophagostomes in Kibale was suggested [43]. Notably, human infections with *O. stephanostomum* were recently demonstrated molecularly in the same locality [9]. However, in a different survey at Kibale, Ghai *et al*. [8] found human infections with *Oesophagostomum* sp., which was different from both *O. stephanostomum* and *O. bifurcum* in ITS2 nucleotide arrangement. This cryptic species was also common in five monkey species there but was not demonstrated in the sympatric chimpanzees [8]. This study could not find this cryptic *Oesophagostomum* sp. in chimpanzees at Bulindi.

Although *O. stephanostomum* and *O. bifurcum* are regarded as zoonotic, only a few records have been made on the human infection with the former species: once in Brazil and several times in Uganda (see reference [1]). The presence of this species in Brazil is curious because it is mostly known from African primates. Moreover, the earlier Uganda cases were regarded as unconvincing [1]. Ghai *et al*. [8] did not find human infection with *O. stephanostomum* during their survey at Kibale, where this nematode was common in chimpanzees and various monkey species. However, Guillot *et al*. [43] and Cibot *et al*. [9] recently reported human cases with *O. stephanostomum* infection in the Sebitoli area of Kibale by DNA amplification. Meanwhile, Makouloutou *et al*. [36] failed to demonstrate *Oesophagostomum* infection in humans residing near Moukalaba-Doudou National Park, Gabon, where *O. stephanostomum* was common in western lowland gorillas. Similarly, while *O. stephanostomum* was demonstrated in bonobos (*Pan paniscus*) at Manzano Forest, Democratic Republic of the Congo, molecular analysis did not reveal *Oesophagostomum* infection in sympatric humans [44]. Possibly, some strains of *O. stephanostomum* are more adaptive to humans, or certain ecological factors provide enhanced conditions for transmission.

In contrast to *O. stephanostomum*, human infection with *O. bifurcum* is well documented, but mostly in northern Togo and northern Ghana [1]. Why the human infection is restricted to the limited areas of these two countries while the parasite is commonly distributed in African primates has been debated for a long time from various viewpoints. Eberhard *et al*. [45], using infective larvae obtained by culture of human faeces in Ghana, found a low level of establishment in experimentally infected rhesus monkeys (*Macaca mulatta*). They regarded that the isolate obtained from humans was less infective to non-human primate hosts, and suggested that *O. bifurcum* found in humans and various monkeys in the same geographical region of northern Ghana and Togo were distinct, and that the human oesophagostomiasis there was not a zoonotic infection acquired from sympatric primates.

Meanwhile, de Gruijter *et al*. [46] made a phylogenetic analysis based on *Cox1* sequences and found no relationship between *O. bifurcum* haplotype groupings and the specific primate host infected. This aspect is also found in this study. In a traditional sense, such an intermingled condition may indicate that the parasites are shared by various host species. Nevertheless, by applying random amplified polymorphic DNA (RAPD) and amplified fragment length polymorphism (AFLP) analyses, it was elucidated that *O. bifurcum* in each host species belongs to genetically distinct groups, supporting the view that *O. bifurcum* is not zoonotic [7,46–49].

It remains unsolved why such host-dependent groups proved by RAPD and AFLP analyses are not reflected in ITS2 genotypes or *Cox1* haplotypes, which have been used generally to reconstruct phylogeny. Possibly, a strain of parasite adapted to a new host species experiences some genetic selection to facilitate further adaptation. If such selection occurs in many loci that are targeted by random amplification, the resulting dendrogram may be host-dependent clusters, regardless of their *Cox1* phylogeny. In order to demonstrate this possibility, it is necessary to identify the loci in the nematode genome that are targeted by the random amplification. Because human infection with *O. bifurcum* is so restricted geographically, it is surmised that the infection was originally derived from non-human primates. Moreover, because rhesus monkeys were susceptible to infection with *O. bifurcum* isolated from humans, albeit at low levels [45], this parasite evidently has potential as a zoonotic pathogen. Transmission to humans could feasibly occur in regions such as Bulindi and elsewhere in Hoima District where chimpanzees and humans have extremely high levels of contact and spatial overlap [12,13,18,22]. Participation of sympatric olive baboons in the maintenance of *O. bifurcum* in Bulindi is currently unknown, but should be an important area of future study.

The *Cox1* phylogenetic tree of *O. bifurcum* also showed peculiar features (figures 2 and 3): some clades (C and D) were composed of haplotypes of worms from geographically distant primates. For example, the haplotypes of worms from the Bulindi chimpanzees and two haplotypes from the yellow baboons of Tanzania formed clade C; other haplotypes from the yellow baboons of Tanzania and three haplotypes from the South African chacma baboon constituted clade D. One haplotype (F) of mona monkeys from Ghana was separated from the other haplotype (E) of mona monkeys and humans. Such a complicated condition could result from rapid geographical dispersal of the worms or the hosts. If various primate species share their *O. bifurcum*infections, the worm haplotypes could spread rapidly over a wide geographical range. Further surveys are necessary to clarify comprehensively the phylogenetic relationships of *O. bifurcum* parasitic in African primates.

## Data accessibility

All DNA sequences reported herein were deposited in DDBJ/EMBL-Bank/GenBank with accession numbers LC063693–LC063722 (ITS2), LC063862–LC063900 (*Cox1*).
